# Versatile mixed-matrix membranes based on AMCD-ZIF and PVC for sustainable water remediation

**DOI:** 10.1039/d5ra06512g

**Published:** 2026-01-05

**Authors:** Fatima Youness, Assil Koubeissy, Rana A. Bilbeisi

**Affiliations:** a American University of Beirut (AUB), Department of Civil and Environmental Engineering Riad El Solh Beirut 1107-2020 Lebanon; b American University of Sharjah, Department of Biology, Chemistry and Environmental Sciences University City Sharjah United Arab Emirates ranabilbeisi@aus.edu

## Abstract

Heavy metal ions and inorganic anions in water sources pose serious environmental and health hazards, necessitating versatile and scalable treatment solutions. In this study, we employ a mixed-matrix membrane based on amine-carboxamide-modified zeolitic imidazolate frameworks (AMCD-ZIF) embedded in electrospun polyvinyl chloride (PVC) nanofibers for the removal of diverse water pollutants. The incorporation of AMCD into PVC nanofibers had been previously optimized for the removal of organic dyes and crude oil from water. Building on this optimized hybrid membrane, we extend its application to the removal of toxic heavy metals and inorganic anions. The functionalized membranes exhibited high adsorption capacities—up to 1666.7 mg g^−1^ for Pb(ii), 400 mg g^−1^ for Ag(i), and 666.7 mg g^−1^ for Cd(ii)—and demonstrated preferential uptake of Pb(ii) in competitive metal systems. Under rapid filtration conditions, the membranes achieved comparable removal efficiencies to batch systems within just 5 minutes for heavy metals. Removal of anionic contaminants such as nitrate, nitrite, sulfate, phosphate, and total nitrogen from real contaminated water collected from Ain El Mreisseh was also investigated. Under rapid filtration conditions, the membranes achieved high removal efficiencies of the anions within 10 minutes: 99.2% for nitrate, 98.6% for nitrite, 95.3% for total nitrogen, 84.6% for sulfate, and 66.8% for phosphate. Additionally, the membranes maintained moderate performance across four regeneration cycles. These results highlight the scalability, reusability, and multifunctionality of the PVC-AMCD membrane as an effective platform for water purification, targeting organic dyes, solvents, crude oil removal, in addition to heavy metals and anionic contaminants investigated in this study.

## Abstract 2

ZIF-based mixed-matrix membrane enables efficient water purification, selectively removing toxic heavy metals, particularly Pb^2+^, and inorganic anions under rapid filtration, demonstrating a versatile, scalable and sustainable treatment platform.

## Graphical abstract

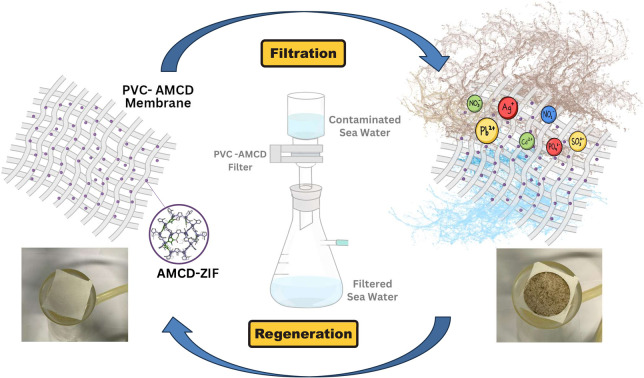

## Introduction

Water pollution poses a global threat to ecosystems, food security, and public health.^1–3^ Contaminants include heavy metals, inorganic anions, and organic pollutants, often originate from industrial, agricultural, and domestic sources,^4–6^ and are increasingly detected in surface water, groundwater, and even treated effluents.^4,7,8^ Sustainable, versatile, and reusable purification technologies are urgently needed to address this challenge.^9^

Among the various classes of water contaminants, heavy metals and inorganic anions are of particular concern due to their chemical stability, bio-accumulative potential, and adverse toxicological effects.^10–12^ Heavy metals such as lead (Pb^2+^), cadmium (Cd^2+^), and silver (Ag^+^) are frequently introduced into aquatic environments through anthropogenic activities including mining, electroplating, battery manufacturing, and industrial discharge.^13–16^ These non-biodegradable metal ions can accumulate in biological tissues, disrupt enzymatic pathways, and are associated with a range of toxic effects, including neurotoxicity, nephrotoxicity, and developmental abnormalities.^6,17,18^ In parallel, inorganic anions such as nitrate (NO_3_^−^), nitrite (NO_2_^−^), sulfate (SO_4_^2−^), and phosphate (PO_4_^3−^) are commonly detected in wastewater as a result of agricultural runoff, excessive fertilizer application, and municipal sewage effluents.^19,20^ Elevated concentrations of these anions can lead to eutrophication, hypoxia, and ecological imbalance, while also posing human health risks, such as methemoglobinemia and gastrointestinal disorders.^21^ Consequently, the effective and simultaneous removal of both heavy metals and anionic pollutants is essential for ensuring water quality compliance and mitigating ecological and human health hazards.^2^

Traditional approaches for the removal of waterborne contaminants, such as chemical precipitation, ion exchange, membrane filtration, and electrochemical treatments, have been widely employed to address both heavy metal ions and inorganic anions.^5,11,16,19^ However, these methods often suffer from significant drawbacks, including high operational costs, energy consumption, secondary waste generation, and limited selectivity, particularly when dealing with complex or dilute aqueous systems.^22,23^ In contrast, adsorption-based technologies have emerged as a more sustainable and adaptable solution for water purification, offering broad-spectrum removal capabilities, operational simplicity, cost-effectiveness, and the potential for regeneration and reuse across multiple treatment cycles.^24,25^

Zeolitic imidazolate frameworks (ZIFs), a subclass of metal–organic frameworks, have emerged as promising materials for environmental remediation due to their high surface area, tunable porosity, and chemical and thermal stability.^26–28^ Among them, ZIF-8 is particularly notable for its robustness in aqueous environments and capacity for functional modification, enabling the selective adsorption of various water pollutants.^29,30^ Recent strategies have integrated ZIFs into polymer matrices, as fillers, to form mixed matrix membranes (MMM) with improved adsorption efficiency, mechanical strength, and antifouling resistance.^31,32^ In this context, electrospun polyvinyl chloride (PVC) has gained interest as a membrane support due to its ease of functionalization, durability, and cost-effectiveness;^25^ however, its intrinsic limitations in adsorption capacity and fouling resistance can be addressed through ZIF modification, enhancing its applicability in water purification.^24,33^

**Scheme 1:**
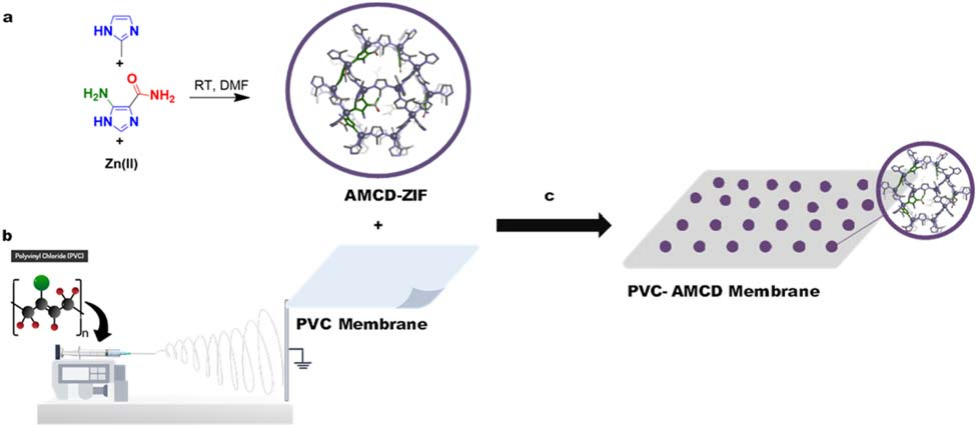
Schematic presentation of the preparation of amine-carboxamide modified ZIF-8 (AMCD-ZIF) (a) and the electrospinning of PVC-membrane (b) followed by the post-synthetic modification of electrospun PVC membrane with AMCD-ZIF (c) in ethanol under basic conditions.

AMCD-ZIF was synthesized using mixed linkers (2-methylimidazole and 4-amino-1*H*-imidazole-5-carboxamide) in DMF. The resulting ZIF was covalently grafted onto the surface of electrospun PVC membranes (Scheme 1). The incorporation of AMCD-ZIF particles as fillers into electrospun PVC membranes to form MMMs was previously reported to be highly effective for the removal of organic dyes and oil–water separation.^24,33^ The successful incorporation and post-functionalization of PVC membranes with AMCD-ZIF have been reported previously.^24,33^ Detailed characterization of the modified electrospun PVC- AMCD membranes is provided in the SI (Fig. S1). Scanning electron microscopy (SEM) image of PVC-AMCD (Fig. S1a) reveals a clear contrast between the smooth PVC fibers (average diameter ≈ 270 nm) and the PVC-AMCD membrane. The latter displays uniformly distributed ZIF nanoparticles anchored onto the fibre surfaces, accompanied by distinct compositional variations that confirm successful surface incorporation. The electrospun fibres of PVC-AMCD retain relatively uniform diameters and continuous morphology, with no evidence of bending, thinning, or bead formation, indicating that the integration of ZIF particles does not compromise the structural integrity of the membrane. FTIR spectra (Fig. S1b) of PVC-AMCD display the expected amide C=O band at ∼1590 cm^−1^ and the N–H vibration at ∼764 cm^−1^, features absent/weaker in neat PVC-M, supporting the chemical grafting of AMCD-ZIF onto the membrane surface. Moreover, the thermal stability of the PVC-AMCD membranes was evaluated using TGA analysis, as shown in Fig. S1c. Notably, the modified membranes exhibited slightly lower thermal stability compared to the pristine PVC membrane. This behaviour can be attributed to the formation of C–N bonds between the PVC fibres and the AMCD-ZIF framework, which decompose at lower temperatures than the C–C bonds of the polymer backbone.^33^ Nevertheless, the PVC-AMCD membrane maintained excellent thermal stability up to 200 °C, highlighting it suitability for industrial applications. Collectively, characterization using SEM, FT-IR, and TGA demonstrate the successful incorporation of AMCD-ZIF while maintaining the structural integrity of the nanofibrous PVC membrane. These earlier studies demonstrated both the efficiency and renderability of the membranes. In the present study, the application of AMCD-ZIF/PVC membranes for water purification was broadened to target a wider range of contaminants, including both heavy metals and inorganic anions. The performance of the AMCD-ZIF/PVC membranes was evaluated using both synthetic solutions and real water samples. Furthermore, the study was extended to explore the scalability of the system, by applying the developed membranes in a larger-scale filtration setup for water treatment. This work presents a practical, and sustainable membrane-based strategy for versatile water purification.

## Experimental procedure

### Materials

Polyvinyl chloride (PVC, Mw = 85 000 g mol^−1^) and silver nitrate (AgNO_3_, MW = 169.87 g mol^−1^) were purchased from Sigma-Aldrich and used without further modification. Ethanol (EtOH, ≥99.8%), dimethylformamide (DMF, ≥99.8%), tetrahydrofuran (THF, ≥99.9%), triethylamine (TEA, ≥99%), zinc acetate dihydrate (Zn(OAc)_2_·2H_2_O), lead nitrate (Pb(NO_3_)_2_, MW = 331.20 g mol^−1^), and cadmium nitrate tetrahydrate (Cd(NO_3_)_2_·4H_2_O, MW = 308.47 g mol^−1^) were obtained from Fisher Scientific and used as received. The ligands 5-amino-1H-imidazole-4-carboxamide (95%) and 2-methylimidazole (99%) were supplied by Acros Organics. Anion detection kits, including NitriVer® 2 (nitrite), NitraVer® 5 (nitrate), PhosVer® 3 (phosphate), and SulfaVer® 4 (sulfate) powder pillows, along with total nitrogen reagent sets (high range, TNT series), were acquired from HACH and used according to the manufacturer's instructions.

### Membrane fabrication and post-modification

A 16 wt% PVC solution was prepared by dissolving 1.936 g of PVC powder in a DMF/THF solvent mixture (7 : 4 v/v), followed by magnetic stirring at 700 rpm for 12 hours at room temperature to obtain a homogeneous, colourless solution. This solution was then loaded into a 25 mL syringe and processed using an electrospinning apparatus. Electrospinning was carried out for 8 hours at room temperature under a constant electric field of 18 kV, with a tip-to-collector distance of 15 cm and a feed rate of 1 mL h^−1^. A commercial nylon screen fabric, characterized by an average pore diameter of 350 µm and a thickness of 215 ± 2 µm, was employed as the collector on a rotating drum operating at 600 rpm. The resulting pristine PVC membrane was subsequently modified by immersing it in an ethanol solution containing AMCD at a 1 : 1 ratio of AMCD to membrane mass. The surface modification procedure, previously optimized and reported in earlier work, was adopted in this study.^33^

### Heavy metal removal measurement

AMCD-ZIF, pristine PVC, and PVC-AMCD membranes were evaluated for their ability to remove Pb(ii), Cd(ii), and Ag(i) ions from aqueous solutions under ambient conditions (neutral pH and room temperature). Each examined adsorbent (10 mg) was immersed in a 150 mg L^−1^ metal ion solution to assess its removal efficiency with a weight-to-volume ratio of 1 : 1. The experiments were conducted under identical conditions, with the samples placed in a shaker operating at 100 rpm and maintained at room temperature. Aliquots were withdrawn at predetermined time intervals of 0.5, 1, 2, and 24 hours, and the solution was subjected to filtration through a 0.45 µm syringe filter to ensure the elimination of solid particulates. The concentration of metal ions in the filtrate was quantified using an iCE 3000 series atomic absorption spectrophotometer, employing an air-acetylene flame as the atomization source. This analytical approach enabled the precise determination of both the removal efficiency (%) and the equilibrium adsorption capacity (*Q_e_*, mg g^−1^). The parameter *Q_e_* denotes the amount of metal ions adsorbed per unit mass of the adsorbent at equilibrium. The corresponding values were calculated using the following equations:1
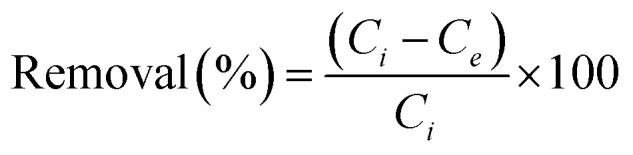
2
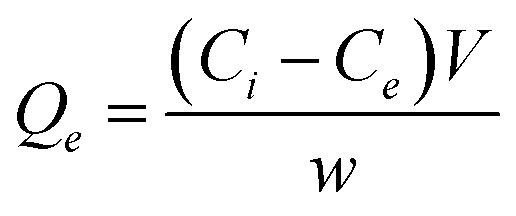
where *C_i_* and *C_e_* (mg L^−1^) are the initial and equilibrium concentrations of metal ion, respectively, *V* is the volume of the solution (L), and *w* is the mass of adsorbents (g).

### Filtration studies

To evaluate the practical performance of the PVC-AMCD membrane under dynamic conditions, two separate filtration experiments were conducted: (1) removal of heavy metals from a synthetic tertiary metal solution, and (2) anion removal from actual wastewater. These experiments serve as a critical step toward translating the membrane's promising properties from controlled laboratory settings to real-world applications. Testing the membrane with complex matrices such as real wastewater not only highlights its chemical and mechanical robustness but also allows for a more accurate assessment of its selectivity, durability, and efficiency under operational conditions. By simulating realistic scenarios, this approach moves beyond proof-of-concept to provide valuable insights into the membrane's scalability, adaptability, and long-term viability for industrial-scale water treatment. Ultimately, these investigations aim to bridge the gap between experimental innovation and functional deployment, supporting the development of next-generation sustainable membranes for broader environmental remediation efforts.

### Heavy metal and anion filtration

Filtration tests were performed using a vacuum-assisted filtration apparatus. Electrospun membranes measuring 4 cm × 4 cm were first pre-wetted with deionized water to ensure uniform permeability, then placed in a standard filter holder. The feed solution consisted of a tertiary mixture of Pb(ii), Cd(ii), and Ag(i), each at an initial concentration of 150 mg L^−1^, prepared in deionized water. A total volume of 50 mL of the solution was poured into the filtration cell and allowed to infiltrate through the membrane for a period of 10 minutes. The filtrate was collected and analysed using AAS to determine the residual concentrations of each metal ion. All filtration experiments were conducted at room temperature, and each test was performed in triplicate to ensure reproducibility. The removal efficiency (%) was calculated based on the concentration difference between the feed and filtrate.

To further demonstrate the versatility of the membranes beyond their previously reported use in dye removal and oil–water separation, as well as their heavy metal adsorption capabilities established in this study, the membranes were evaluated for anion removal from raw seawater wastewater. The collected wastewater was first analysed to identify its content of common anionic pollutants, including sulfate (SO_4_^2−^), phosphate (PO_4_^3−^), nitrate (NO_3_^−^), nitrite (NO_2_^−^), and total nitrogen (TN). These anions were quantitatively assessed before and after membrane treatment using UV-Vis spectrophotometric techniques. The filtration procedure follows the same as for the metal ions. A 50 mL aliquot of raw wastewater was filtered through the 4 cm × 4 cm PVC-AMCD membrane under vacuum after leaving it in contact for 10 minutes. The filtrate was then analysed for residual anion content. The anion concentrations were measured using a DR3900 Laboratory VIS Spectrophotometer (HACH) according to the manufacturer's reagent-based protocols. For each test, a 10 mL water sample was placed in a clean glass vial and mixed with the corresponding reagent powder pillow: NitriVer® 2 for nitrite, NitraVer® 5 for nitrate, SulfaVer® 4 for sulfate, and PhosVer® 3 for phosphate. The mixtures were allowed to react for specific durations, 20 minutes for nitrite, 5 minutes for nitrate and sulfate (after shaking nitrate for 1 minute), and 2 minutes for phosphate. Untreated water samples were used as blanks in all cases, with the instrument first zeroed before measurement. The spectrophotometer programs were selected according to the analyte, and absorbance was recorded at 371 nm (nitrite), 355 nm (nitrate), 680 nm (sulfate), and 490 nm (phosphate). Final concentrations were reported in mg L^−1^. Total nitrogen concentrations were determined using HACH's High Range (HR) TNT reagent set, following the manufacturer's instructions. After sample digestion and reagent addition, the TNT vial was inserted into the DR3900, which automatically recognized the method *via* the 2D barcode printed on the vial label. The spectrophotometer then performed the measurement and reported the nitrogen concentration in mg L^−1^ without requiring manual program selection.

This filtration test was designed to mimic pressure-driven flow conditions commonly found in portable filtration systems and industrial water treatment operations.^34,35^ It aimed to determine whether the high adsorption efficiencies observed under batch conditions could be maintained during continuous flow applications.

## Results and discussion

### Removal efficiency in homo-ionic systems

**Fig. 1:**
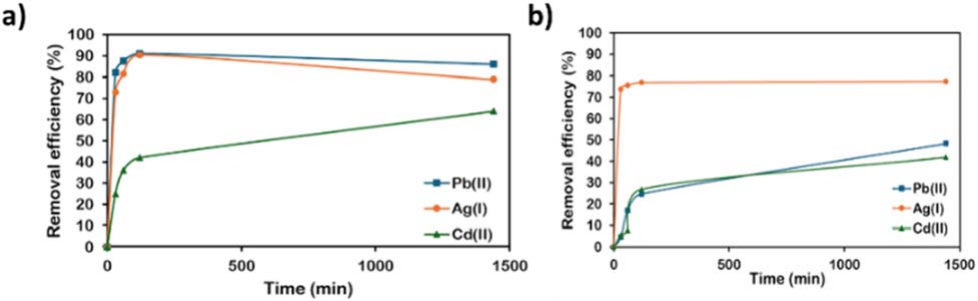
Heavy metals removal efficiency for: (a) AMCD-ZIF and (b) PVC-AMCD membrane.

The heavy metal removal performance of AMCD-ZIF particles, both in powder form and when incorporated into the PVC membrane, was evaluated at neutral pH and room temperature. In each case, 10 mg of AMCD and PVC-AMCD were dispersed in a 150 mg L^−1^ metal ion solution to assess their adsorption behaviour. Kinetic experiments demonstrated a high adsorption affinity of AMCD, particularly toward lead(ii) and silver(i), with removal efficiencies of 91.1% and 90.5%, respectively, within the first two hours. Cadmium(ii), by contrast, exhibited a lower removal efficiency of 42% at the same time point. After 24 hours, the final removal efficiencies slightly declined to 86.0% for Pb(ii), 80.0% for Ag(i), and 64.0% for Cd(ii), likely due to surface saturation (Fig. 1a). The comparatively lower removal of cadmium is consistent with its weaker interaction with the amine groups in the AMCD structure.^36,37^

The performance of 10 mg of PVC-AMCD membrane was similarly evaluated, revealing notable improvements in metal removal over the pristine PVC membrane (Fig. S2). After 24 hours, the modified membrane achieved maximum removal efficiency of 48.3% for Pb(ii), 77.5% for Ag(i), and 41.9% for Cd(ii). Kinetic profiles revealed that silver(i) exhibited the fastest adsorption rate among the three metals, attributed to the strong interaction between Ag(i) ions and the lone electron pairs on the amino group (nitrogen atoms) of the AMCD structure.^38,39^ Although the removal capacities were lower than those of AMCD powder, the incorporation of AMCD into the PVC matrix significantly enhanced the membrane's adsorption performance compared to pristine PVC, which exhibited poor removal rates for all tested metals, with improvements of 47.4% for Pb(ii), 84.7% for Ag(i), and 83.2% for Cd(ii).

The evaluation of AMCD and PVC-AMCD highlights the superior removal efficiency of AMCD in its powdered form, attributed to the greater availability of active adsorption sites. The reduced efficiency observed in the PVC-AMCD membrane is likely due to the relatively lower incorporation and restricted accessibility of AMCD within the polymer matrix.^24^ Despite this, PVC-AMCD offers notable advantages in handling, mechanical stability, and scalability, making it a practical option for large-scale applications (which is explored and reported in this study in Section 3.5). Overall, these findings underscore the strong potential of AMCD and its PVC-AMCD for effective heavy metal remediation.

### Adsorption kinetics and isotherm

Kinetic studies were conducted to assess the rate of adsorption of Pb(ii), Ag(i), and Cd(ii) on AMCD particles and PVC-AMCD membranes.^40^ The adsorption kinetics followed the pseudo-second-order model with high correlation coefficients (*R*^2^ > 0.98) across all systems, indicating that the adsorption process is mainly achieved by chemisorption.^25,40^ The kinetic rate constant (*K*_2_), equilibrium adsorption capacities (*q*_*e*_), and the correlation coefficients (*R*^2^) values are summarized in Table S2. As shown in Fig. S3a–f, Pb(ii) and Ag(i) demonstrated more rapid uptake, over 2 hours, compared to Cd(ii), which is attributed to their stronger affinity for the nitrogen-containing functional groups present in AMCD.^41–43^ Cd(ii) exhibited slower adsorption kinetics, indicating a less favourable interaction with the adsorbent surface.^36,39^ These trends were consistent for both AMCD and PVC-AMCD, suggesting that the incorporation of AMCD into the polymer matrix preserved the accessibility and reactivity of its active adsorption sites.^24^

**Fig. 2:**
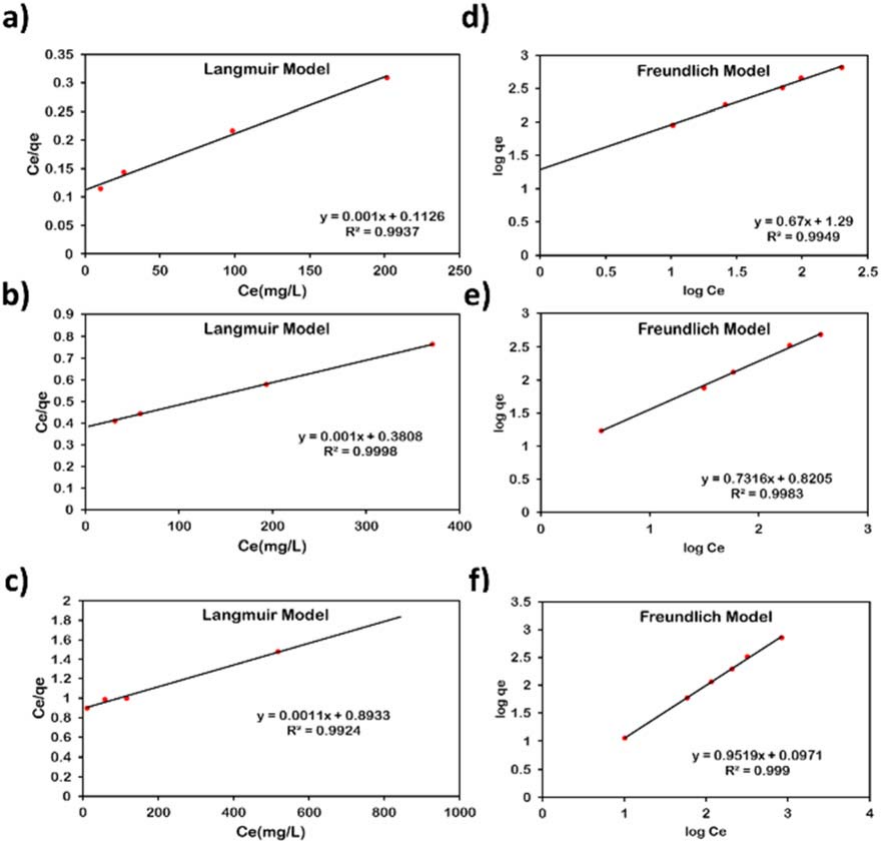
Langmuir isotherm model of AMCD-ZIF for: (a) Pb(ii), (b) Ag(i), and (c) Cd(ii); Freundlich isotherm model of AMCD-ZIF for: (d) Pb(ii), (e) Ag(i), and (f) Cd(ii).

**Fig. 3:**
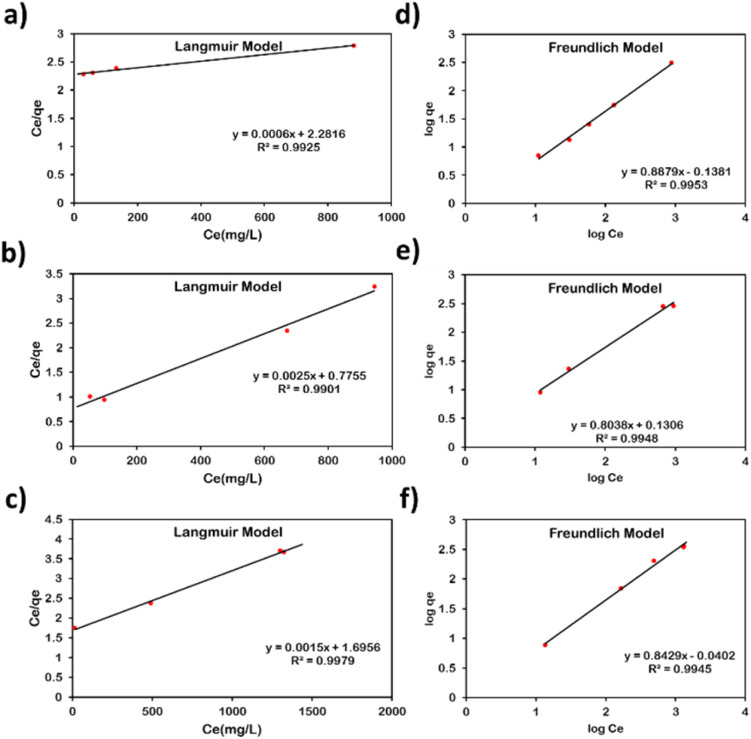
Langmuir isotherm model of PVC-AMCD membrane for: (a) Pb(ii), (b) Ag(i), and (c) Cd(ii); Freundlich isotherm model of PVC-AMCD membrane for: (d) Pb(ii), (e) Ag(i), and (f) Cd(ii).

Moreover, the equilibrium adsorption data were further analysed to investigate the adsorption mechanism and measure the maximum adsorption capacity of the adsorbents towards the selected metals.^44^ The adsorption isotherm data of the three metals fitted well to Langmuir and Freundlich models, with *R*^2^ values exceeding 0.99 (Fig. 2 and Fig. 3), indicating that both models describe the adsorption behaviour. The Langmuir model assumes monolayer adsorption on homogeneous sites, while the Freundlich model accounts for multilayer adsorption on heterogeneous surfaces.^29,42,45,46^ The data suggests a mixed adsorption process involving interactions at both homogeneous and heterogeneous sites.^45,47–49^

The maximum adsorption capacities (*q*_m_) obtained from the Langmuir model were 1000 mg g^−1^ for Pb(ii), 1000 mg g^−1^ for Ag(i), and 909.1 mg g^−1^ for Cd(ii) on AMCD, and 1666.7 mg g^−1^, 400 mg g^−1^, and 666.7 mg g^−1^ for the same metals on PVC-AMCD, respectively (Table 1). These values confirm the high affinity and capacity of both adsorbents for heavy metal removal. Notably, the adsorption capacity of Pb(ii) on PVC-AMCD exceeded that of AMCD alone. This enhancement is unexpected compared to the trends observed for Ag(i) and Cd(ii), and may be attributed to the moderate affinity of pristine PVC toward Pb(ii), as also reported in our previous work.^25^ The pristine PVC membrane achieved up to 25% removal efficiency for Pb(ii), unlike its negligible affinity for other metal ions (Table S1). This suggests that the PVC contributes to Pb(ii) adsorption, leading to a synergistic effect when combined with AMCD in the mixed matrix membrane. The combined active sites on AMCD and the PVC nanofibers likely resulted in the elevated Pb(ii) uptake observed in the functionalized system.

**Table 1:** Adsorption isotherm model constants derived from Langmuir and Freundlich isotherms

Isotherm model	Parameters	AMCD			PVC-AMCD		
		Pb(ii)	Ag(i)	Cd(ii)	Pb(ii)	Ag(i)	Cd(ii)
Langmuir model	*R* ^2^	0.9937	0.9998	0.9924	0.9925	0.9901	0.9979
	*q*_max_ (mg g^−1^)	1000	1000	909.1	1666.7	400	666.7
	*K*_L_ (L mg^−1^)	0.0089	0.0026	0.0011	0.00024	0.0013	0.00059
	*R*_L_	0.12–0.53	0.24–0.95	0.47–0.98	0.62–0.98	0.36–0.89	0.55–0.99
Freundlich model	*R* ^2^	0.9949	0.9983	0.999	0.9953	0.9948	0.9945
	*K*_F_ (mg g^−1^)	19.50	6.61	1.25	0.73	1.35	0.91
	1/*n*	0.67	0.73	0.95	0.89	0.80	0.84
	*n*	1.49	1.37	1.05	1.12	1.25	1.19

Moreover, the Langmuir separation factor (*R*_L_) ranged from 0.12–0.99, indicating that the adsorption of the metal ions onto AMCD and PVC-AMCD is favorable.^50^ In parallel, the Freundlich exponent (*n*) ranged between 1.05–1.49, supporting a favourable adsorption and suggesting a heterogeneous distribution of surface sites of the adsorbent.^50^

Overall, the kinetics and isotherm analyses confirm that metal ion adsorption onto AMCD and PVC-AMCD occurs through a combination of monolayer chemisorption and multilayer physisorption. This mixed mechanism is governed by both the strength of interaction between the metal ions and the functional groups on AMCD, as well as the surface heterogeneity introduced by the functionalized membrane.^45,47–49,51^

### Competitive adsorption in tertiary metal systems

**Fig. 4:**
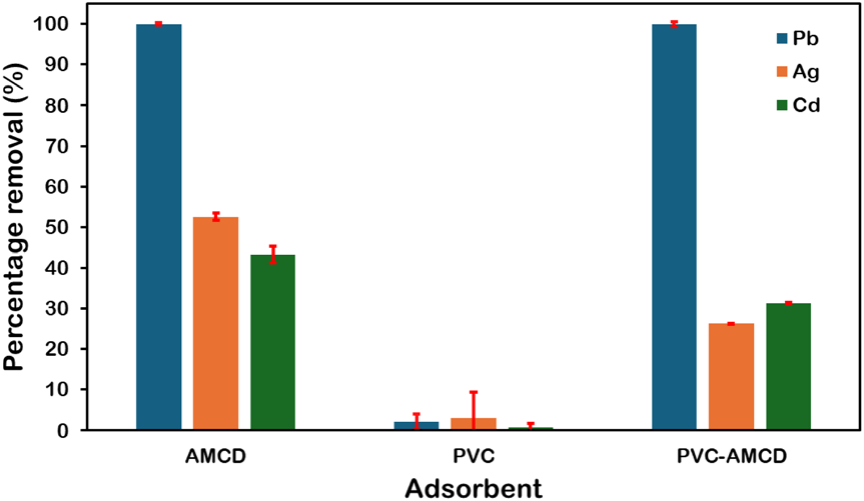
Removal efficiencies of 10 mg of AMCD, PVC, and PVC-AMCD membranes in a 150 mg L^−1^ tertiary metal ions system after 24 hours.

A tertiary system containing 150 mg L^−1^ of Pb(ii), Cd(ii), and Ag(i) was studied over 24 hours to mimic realistic water conditions involving multiple metal contaminants and to evaluate the competitive adsorption behaviour and selectivity of AMCD, pristine PVC, and PVC-AMCD membranes. As shown in Fig. 4, AMCD particles demonstrated the highest removal efficiency, achieving nearly 98% for Pb(ii), 53% for Ag(i), and 45% for Cd(ii), highlighting their strong affinity for heavy metals. In contrast, unmodified PVC showed negligible adsorption across all metals (below 5%), confirming its chemically inert nature and lack of functional groups. Notably, the incorporation of AMCD into PVC fibres significantly enhanced membrane performance, with the resulting PVC-AMCD membrane achieving nearly complete removal of Pb(ii) (∼99%), along with moderate removal of Ag(i) (∼26%) and Cd(ii) (∼30%).

Pb(ii) exhibited the highest removal efficiency in the competitive system, a result attributed to its relatively higher electronegativity, smaller hydration radius, and strong coordination sphere.^52^ Previous studies have shown that heavy metal cations with higher electronegativity, like Pb(ii), are more likely to outcompete ions with lower electronegativity for adsorption sites.^25^ Additionally, Pb(ii)'s relatively smaller hydration radius and favourable coordination behaviour can further enhance its selective uptake over Ag(i) and Cd(ii) in multi-metal systems.^25,53,54^ Thus, PVC-AMCD membranes demonstrated effective and selective removal behaviour even under competitive adsorption, maintaining a clear preference for Pb(ii), and confirming their potential for advanced wastewater treatment applications.

### Regeneration and reusability of PVC-AMCD

The regeneration and reusability of the PVC-AMCD membrane were evaluated through successive adsorption–desorption cycles using 0.1 M HCl solution (pH ≈ 4), focusing on Pb(ii) and Ag(i) removal. After each filtration cycle, the membrane was soaked in acid for one hour to desorb the adsorbed metals. As shown in Fig. S4, the PVC-AMCD membrane maintained moderate reusability, with Pb(ii) removal efficiency decreased by 49%, while Ag(i) removal dropped by 32% over four regeneration cycles. SEM analysis (Fig. S5) revealed a noticeable reduction in AMCD particles attached to the PVC nanofibers after repeated regeneration cycles, indicating potential loss of functional material and reduced membrane stability. Despite the decrease in efficiency compared to pristine AMCD, the PVC-AMCD membrane offers reasonably good efficiency, reusability, and stability, essential for practical water treatment applications.

In earlier studies on oil and dye removal, the PVC-AMCD membrane maintained performance over five cycles using only mechanical or simple solvent washing.^24,33^ However, in this study, the strong coordination between metal ions and the ZIF active sites necessitated a harsher regeneration step. While the strong acid HCl proved effective in desorbing the metals, the process likely contributed to ZIF detachment. Therefore, future efforts should focus on optimizing the regeneration protocol using milder desorbing agents that preserve the membrane's functional integrity while maintaining high reusability for metal removal.

### Rapid filtration performance of mixed contaminants

To assess real-world applicability, the PVC-AMCD membrane was tested under dynamic conditions for removing (1) heavy metals from a synthetic tertiary metal solution and (2) anions from real seawater wastewater. These rapid filtration experiments were conducted using a 4 × 4 cm membrane (30 mg) operated under vacuum for 5 and 10 minutes, respectively (Fig. S6). The tests simulate conditions suitable for large-scale implementation and highlight the membrane's robustness, selectivity, and efficiency in complex matrices. This marks a key step in translating lab-scale innovation to practical environmental remediation. Ultimately, the findings support the membrane's scalability and potential for sustainable water treatment applications.

In the case of heavy metals, the membrane demonstrated selective removal efficiencies from a 150 mg L^−1^ metal solution with Pb(ii) showing the highest uptake (∼58%), followed by Cd(ii) (∼36%) and Ag(i) (∼24%) within just 5 minutes of contact time (Fig. S7). The relatively high electronegativity and favourable coordination behaviour of Pb(ii) likely contributed to its preferential adsorption, even under limited contact time. Ag(i) showed lower removal efficiency in the rapid filtration setup, which may be attributed to kinetic limitations and competition among metal ions during the short contact time. Cd(ii) demonstrated moderate removal, further confirming the membrane's ability to adsorb a broad range of heavy metals.

**Fig. 5:**
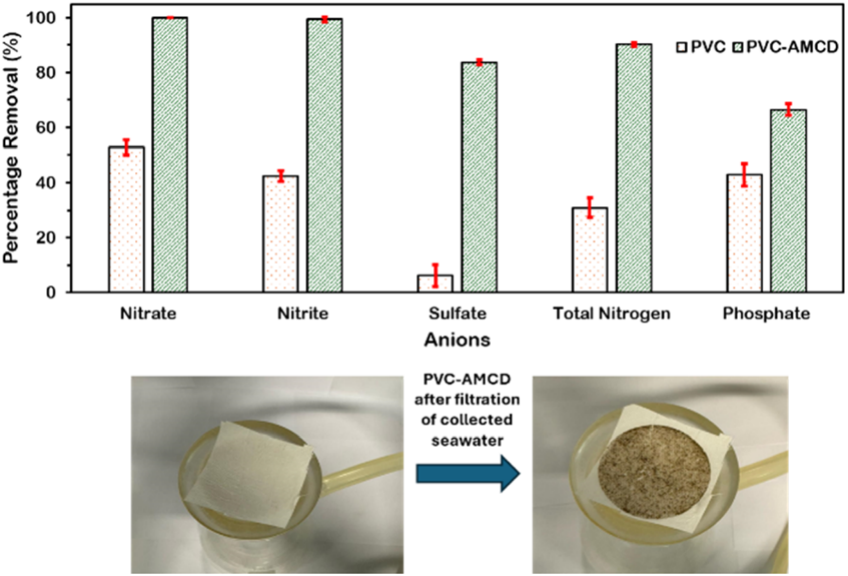
Percentage removal of anions from wastewater collected from Ain El Mreisseh using pristine PVC and PVC-AMCD membranes.

In parallel, the same filtration setup was applied to real seawater collected from Ain El Mreisseh, Beirut, to assess the membrane's performance in anion removal. The selected anions: nitrate (NO_3_^−^) nitrite (NO_2_^−^), total nitrogen (TN), sulfate (SO_4_^−2^), and phosphate (PO_4_^−3^), were chosen due to their increasing presence in municipal and industrial wastewater effluents, as well as their significant role in promoting eutrophication, algal blooms, and the overall degradation of aquatic ecosystems.^19,55^ Hence, efficient removal of these anions plays a crucial role in controlling eutrophication and improving the water quality. A 30 mg PVC-AMCD membrane with dimensions of 4 × 4 cm achieved notable removal efficiencies during anion filtration within a 10 minutes contact time, including 99.2% for nitrate, 98.6% for nitrite, 95.3% for total nitrogen, 84.6% for sulfate, and 66.8% for phosphate (Fig. 5). Compared to pristine PVC, the enhanced performance was attributed to AMCD surface functionalization, which led to a significant improvement in anion removal by 47.3% for nitrate, 57.4% for nitrite, 61.6% for total nitrogen, 92.8% for sulfate, and 34.0% for phosphate. The removal of anions is mainly governed by electrostatic attraction and hydrogen bonding.^56^ Although the alkaline pH of seawater (∼8) reduces the protonation of amine groups, a fraction of surface sites may remain positively charged. These residual charges can facilitate electrostatic interactions with anionic species such as sulfate and phosphate.^56–59^ Moreover, the amine and amide groups within AMCD can form stable hydrogen bonds with oxygen-rich (oxoanions) anions,^60^ which is pH-independent and effective even under high-salinity conditions.^59^

Together, these findings validate the versatility of the PVC-AMCD membrane in removing both heavy metals and anions within short contact times, demonstrating its potential as a scalable and multifunctional platform for rapid and efficient water purification.

## Conclusion

In this study, the mixed matrix membrane prepared through the incorporation of amine-carboxamide-modified zeolitic imidazolate framework (AMCD) onto electrospun polyvinyl chloride (PVC) membrane was investigated for the removal of a selection of heavy metals and inorganic anionic pollutants from water. Comparative adsorption analyses between powder AMCD ZIF, pristine PVC, and PVC-AMCD membranes demonstrated a significant enhancement in removal efficiency of heavy metals and anions from water using AMCD ZIF and PVC-AMCD membrane.

Heavy metals adsorption experiments conducted on AMCD powder and PVC-AMCD membranes revealed that the adsorption process followed the pseudo-second-order kinetic model and fit well to both Langmuir and Freundlich isotherms, indicating a combined monolayer-multilayer adsorption mechanism. The PVC-AMCD membrane exhibited a maximum adsorption capacity of 1666.7 mg g^−1^ for Pb(ii), demonstrating improved performance relative to a number of earlier polymer nanofiber adsorbents (Table S3). It also achieved removal efficiencies of 48.3% for Pb(ii), 77.5% for Ag(i), and 41.9% for Cd(ii), representing improvements of 47.4%, 84.7%, and 83.2% respectively over pristine PVC. In mixed-metal systems, the membrane showed a strong preference for Pb(ii) over Ag(i) and Cd(ii), attributed to its favourable coordination behaviour and ionic characteristics.

Beyond heavy metal removal, the AMCD-functionalized membrane effectively removed common anions from real seawater, achieving 99.2% for nitrate, 98.6% for nitrite, 95.3% for total nitrogen, 84.6% for sulfate, and 66.8% for phosphate within short contact times. Compared to pristine PVC, these removals represent improvements of up to 92.8%, highlighting the enhanced performance achieved through surface functionalization. Furthermore, the membrane maintained moderate performance over multiple regeneration cycles, with moderate loss in efficiency due to gradual detachment of AMCD particles. This study introduces a versatile nanofibrous membrane with high selectivity and broad-spectrum removal capacity, suitable for rapid and scalable water treatment. By reporting this work, we demonstrate the effectiveness of a single membrane system in removing a diverse range of pollutants, including oils, heavy metals, and anions, from contaminated water.

## Conflicts of interest

“There are no conflicts to declare”.

## Supplementary Material

RA-016-D5RA06512G-s001

## Data Availability

The authors of this manuscript declare that the experimental data supporting this article has been included as part of the supplementary information (SI). Supplementary information: for general methods, supplementary details on the removal efficiency, sorption isotherms and kinetics, ion selectivity, and regeneration of the adsorbents. See DOI: https://doi.org/10.1039/d5ra06512g.

## References

[cit1] du Plessis A. (2022). One Earth.

[cit2] Ejiohuo O., Onyeaka H., Akinsemolu A., Nwabor O. F., Siyanbola K. F., Tamasiga P., Al-Sharify Z. T. (2025). Water Biol. Secur..

[cit3] Babuji P., Thirumalaisamy S., Duraisamy K., Periyasamy G. (2023). Water.

[cit4] Zhang P., Yang M., Lan J., Huang Y., Zhang J., Huang S., Yang Y., Ru J. (2023). Toxics.

[cit5] Yazdi F., Anbia M., Sepehrian M. (2023). Carbohydr. Polym..

[cit6] Briffa J., Sinagra E., Blundell R. (2020). Heliyon.

[cit7] Li P., Karunanidhi D., Subramani T., Srinivasamoorthy K. (2021). Arch. Environ. Contam. Toxicol..

[cit8] Nishmitha P. S., Akhilghosh K. A., Aiswriya V. P., Ramesh A., Muthuchamy M., Muthukumar A. (2025). J. Hazard. Mater. Adv..

[cit9] Qi Y., He K. (2025). Water.

[cit10] Hama Aziz K. H., Mustafa F. S., Omer K. M., Hama S., Hamarawf R. F., Rahman K. O. (2023). RSC Adv..

[cit11] Meftah S., Meftah K., Drissi M., Radah I., Malous K., Amahrous A., Chahid A., Tamri T., Rayyad A., Darkaoui B., Hanine S., El-Hassan O., Bouyazza L. (2025). Discov. Sustain..

[cit12] Ammar M., Yousef E., Ashraf S., Baltrusaitis J. (2024). Separations.

[cit13] Reda A., El-Demerdash A.-G., Sadik W., El-Rafey E., Shoeib T. (2025). Appl. Water Sci..

[cit14] Jadaa W., Mohammed H. K. (2023). J Ecol Eng.

[cit15] Kumar M., Seth A., Singh A. K., Rajput M. S., Sikandar M. (2021). Environ. Sustain. Indic..

[cit16] Bharti S. (2025). Int. J. Environ. Sci. Technol..

[cit17] Mitra S., Chakraborty A. J., Tareq A. M., Emran T. B., Nainu F., Khusro A., Idris A. M., Khandaker M. U., Osman H., Alhumaydhi F. A., Simal-Gandara J. (2022). J. King Saud Univ. - Sci..

[cit18] Tchounwou P. B. , Yedjou C. G., Patlolla A. K. and Sutton D. J., in Molecular, Clinical and Environmental Toxicology, ed. A. Luch, Springer Basel, Basel, 2012, 3, pp. 133–164

[cit19] Usman M. O., Aturagaba G., Ntale M., Nyakairu G. W. (2022). Water Sci. Technol..

[cit20] Karthikeyan P., Meenakshi S. (2020). Sustain. Mater. Technol..

[cit21] Li J., Jin Q., Liang Y., Geng J., Xia J., Chen H., Yun M. (2022). Int. J. Environ. Res. Public. Health.

[cit22] Fei Y., Hu Y. H. (2023). Chemosphere.

[cit23] Ayach J., El Malti W., Duma L., Lalevée J., Al Ajami M., Hamad H., Hijazi A. (2024). Polymers.

[cit24] Jaafar A., El-Husseini S., Platas-Iglesias C., Bilbeisi R. A. (2022). J. Environ. Chem. Eng..

[cit25] Youness F., Jaafar A., Tehrani A., Bilbeisi R. A. (2022). RSC Adv..

[cit26] Ma D., Cheng Z., Yuan Y., Chaemchuen S. (2024). J. Environ. Chem. Eng..

[cit27] Yuan Y., Li X., Sun X., Sun Y., Yang M., Liu B., Yang D., Li H., Liu Y. (2025). Nano Energy.

[cit28] Naseraei M. M., Adeli H., Nabavi S. R., Salimi-Kenari H., Mansour R. N., Sarkati A. G. (2025). Int. J. Biol. Macromol..

[cit29] Yang W., Kong Y., Yin H., Cao M. (2023). J. Solid State Chem..

[cit30] di Nicola N., Di Pelino M., Foschi M., Passalacqua R., Lazzarini A., Ruggieri F. (2025). Molecules.

[cit31] Chen Y., Jiang L. (2021). Appl. Water Sci..

[cit32] Khan W. S. , Khan N. U. and Janjua M. M., in 2019 Advances in Science and Engineering Technology International Conferences (ASET), 2019, pp. 1–6

[cit33] Youness F., AlDhawi Z. A., Abdulhamid M. A., Bilbeisi R. A. (2025). Sep. Purif. Technol..

[cit34] Van Der Bruggen B., Vandecasteele C., Van Gestel T., Doyen W., Leysen R. (2003). Environ. Prog..

[cit35] Obotey Ezugbe E., Rathilal S. (2020). Membranes.

[cit36] Wang K., Gu J., Yin N. (2017). Ind. Eng. Chem. Res..

[cit37] Jaafar A., Youness F., Bilbeisi R. A. (2024). Appl. Organomet. Chem..

[cit38] Wang Q., Li M., Xi M., Zhao M., Wang X., Chen X., Ding L. (2024). Toxics.

[cit39] Zhang Q., Wang Y., Liu H., Zhang H., Wu Z., Zhang S., Li J., Han W., Ye X. (2025). Sep. Purif. Technol..

[cit40] Chen P., Wang Y., Zhuang X., Liu H., Liu G., Lv W. (2023). J. Environ. Sci..

[cit41] Liao W., Liu Y., Liu Y. (2024). Sep. Purif. Technol..

[cit42] Shao Z., Di K., Ding L., You F., Fan C., Wang K. (2025). Anal. Chim. Acta.

[cit43] He Y., Liu Q., Hu J., Zhao C., Peng C., Yang Q., Wang H., Liu H. (2017). Sep. Purif. Technol..

[cit44] de Mello J. R., Machado T. S., Crestani L., Alessandretti I., Marchezi G., Melara F., Mignoni M. L., Piccin J. S. (2022). Heliyon.

[cit45] Foo K. Y., Hameed B. H. (2010). Chem.–Eng. J..

[cit46] Kalam S., Abu-Khamsin S. A., Kamal M. S., Patil S. (2021). ACS Omega.

[cit47] Wang X., Zhou Y., Men J., Liang C., Jia M. (2023). Inorganica Chim. Acta.

[cit48] Liu S., Huang J., Shi L., He W., Zhang W., Li E., Zhang C., Pang H., Tan X. (2024). Environ. Pollut..

[cit49] Ngah W. S. W., Fatinathan S. (2010). J. Environ. Manage..

[cit50] Desta M. B. (2013). J. Thermodyn..

[cit51] Bulut Y., Tez Z. (2007). J. Environ. Sci..

[cit52] Fan X., Liu H., Anang E., Ren D. (2021). Materials.

[cit53] Huda B. N., Wahyuni E. T., Mudasir M. (2023). South Afr. J. Chem. Eng..

[cit54] Bashir A., Manzoor T., Malik L. A., Qureashi A., Pandith A. H. (2020). ACS Omega.

[cit55] Kasiński S., Kowal P., Czerwionka K. (2025). Materials.

[cit56] Zhao C., Du Y., Zhang J., Mi Y., Su H., Fei T., Li S., Pang S. (2020). ACS Appl. Mater. Interfaces.

[cit57] Lin K.-Y. A., Chen S.-Y., Jochems A. P. (2015). Mater. Chem. Phys..

[cit58] Amin N. A. A. M., Mokhter M. A., Salamun N., Wan Mahmood W. M. A. (2021). Membranes.

[cit59] Wang Y., Zhao W., Qi Z., Zhang L., Peng Y. (2020). Sci. Total Environ..

[cit60] Isaeva V. I., Vedenyapina M. D., Kurmysheva A. Yu., Weichgrebe D., Nair R. R., Nguyen N. P. T., Kustov L. M. (2021). Molecules.

