# Supplementary material for: Versatile mixed-matrix membranes based on AMCD-ZIF and PVC for sustainable water remediation
Source: RSC Adv. 2026 Jan 5;16(1):945–52. doi: 10.1039/d5ra06512g (PMC12766271; doi:10.1039/d5ra06512g)
Supplement: RA-016-D5RA06512G-s001 [file RA-016-D5RA06512G-s001.pdf]

Supplementary Information (SI) for RSC Advances.
This journal is © The Royal Society of Chemistry 2025

Supporting Information

Versatile Mixed-Matrix Membranes Based on AMCD-ZIF and PVC for Sustainable Water Remediation

Fatima Youness[1], Assil Koubeissy[1], Rana Bilbeisi[2*]

Table of Contents

List of Figures 2
List of Tables 2
1. AMCD-ZIF incorporation and structural integrity of PVC-AMCD 3
2. Removal efficiency of AMCD, PVC, and PVC-AMCD for heavy metals 3
3. Adsorption kinetics 4
4. Adsorption isotherm 5
4.1. Langmuir isotherm model 6
4.2. Freundlich isotherm model 7
5. Regeneration and reusability of PVC-AMCD membrane 7
6. Rapid filtration performance of mixed contaminants 8
7. Comparative analysis of adsorption capacities towards lead(II) ions 9
8. References 10

List of Figures

Figure S1: Characterization of PVC-AMCD supporting the incorporation of AMCD-ZIF into the electrospun PVC membrane; a) SEM image with a water contact angle insert, b) Thermogravimetric analysis of PVC-AMCD relative to the pristine ZIF and membrane, and c) FTIR of the PVC-AMCD relative to the pristine membrane. 3
Figure S2: Removal efficiency of AMCD, PVC, and PVC-AMCD for: a) lead(II), b) silver(I), and c) cadmium(II) from water..3
Figure S3: The pseudo-second-order kinetic plot for the adsorption of: a) ([Ag(I)] = 150 mg L−1) on a 10 mg AMCD, b) ([Pb(II)] = 150 mg L−1) on a 10 mg AMCD, c) ([Cd(II)] = 150 mg L−1) on a 10 mg AMCD, d) ([Ag(I)] = 150 mg L−1) on a 10 mg PVC-AMCD membrane, e) ([Pb(II)] = 150 mg L−1) on a 10 mg PVC-AMCD membrane, f) ([Cd(II)] = 150 mg L−1) on a 10 mg PVC-AMCD membrane. 5
Figure S4: Reusability of PVC-AMCD for the adsorbed amount of Ag(I) and Pb(II). 7
Figure S5: SEM images of PVC-AMCD membrane before and after regeneration. 8
Figure S6: Filtration setup and PVC-AMCD membrane after filtration of multi-metal solution ..8
Figure S7: Percentage removal of 4x4 cm PVC-AMCD membrane during the filtration of a multi-metal solution. The solution was left in contact with the membrane for 10 minutes 9
Figure S8: Real wastewater: a) before filtration and b) after filtration using PVC-AMCD membrane 9

List of Tables

Table S1: Removal efficiencies of AMCD, pristine PVC, and PVC-AMCD membrane in both homoionic and tertiary systems 4
Table S2: Parameters in the kinetic models of pseudo-second order. 4
Table S3*:* Comparison of maximum lead(II) adsorption capacity qmax (mg g-1) of PVC-AMCD membrane with previously reported MOF/ZIF-based membranes 9

1. AMCD-ZIF incorporation and structural integrity of PVC-AMCD

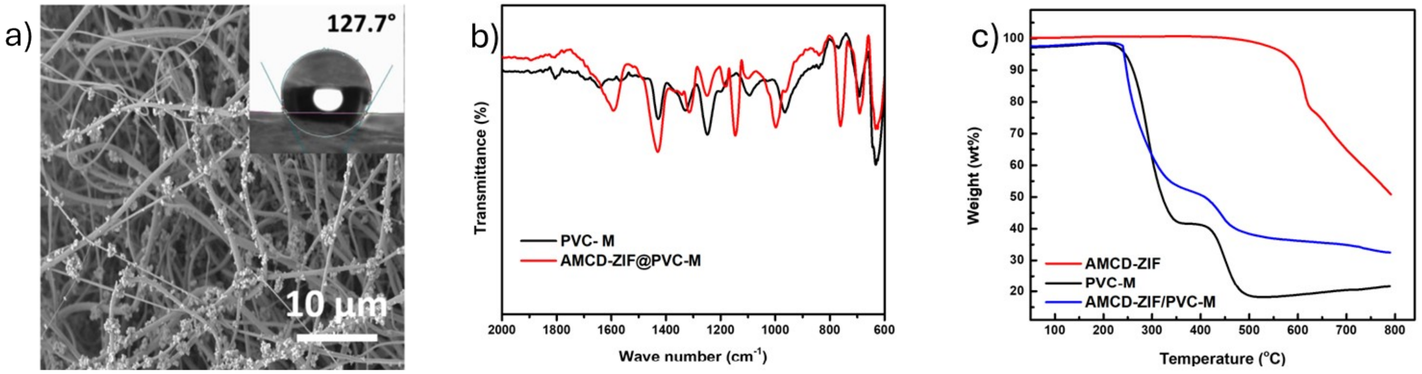

Figure S1: Characterization of PVC-AMCD supporting the incorporation of AMCD-ZIF into the electrospun PVC membrane; a) SEM image with a water contact angle insert, b) Thermogravimetric analysis of PVC-AMCD relative to the pristine ZIF and membrane, and c) FTIR of the PVC-AMCD relative to the pristine membrane.

2. Removal efficiency of AMCD, PVC, and PVC-AMCD for heavy metals

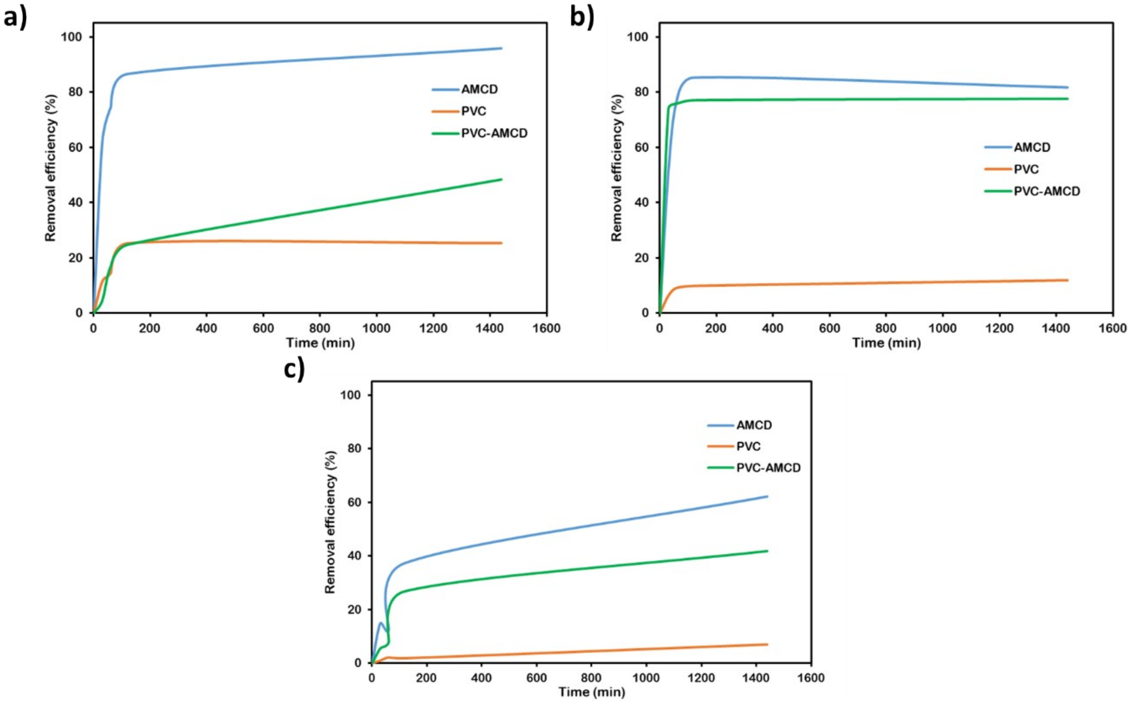

Figure S2: Removal efficiency of AMCD, PVC, and PVC-AMCD for: a) lead(II), b) silver(I), and c) cadmium(II) from water.

Table S1: Removal efficiencies of AMCD, pristine PVC, and PVC-AMCD membrane in both homoionic and tertiary systems

Ionic system	Metal ions	Removal efficiency (%) after 24 hrs

		AMCD	PVC	PVC-AMCD
**Homoionic system**	Pb(II)	86.0	25.4	48.3
	Ag(I)	78.8	11.9	77.5
	Cd(II)	63.9	7.0	41.9
**Tertiary system**	Pb(II)/Ag(I)/Cd(II)	100/52.5/43.3	2.1/2.9/0.85	100/26.4/31.3

3. Adsorption kinetics

The kinetic data were fitted with the pseudo-second-order kinetic model using the following equation (**EQN S1**):[1]

$$\frac{t}{qt} = \frac{1}{K_2 q_e^2} + \frac{t}{q_e} \qquad \textbf{(S1)}$$

Where qe (mg/g) and qt (mg/g) are the amount of metal ions adsorbed at equilibrium and at time t (min), respectively; t (min) is the adsorption time and K_2 (g/mg/min) is the pseudo-second-order adsorption rate constant.

Table S2: Parameters in the kinetic models of pseudo-second order.

	**AMCD**			**PVC-AMCD**		
**Parameter**	*Ag(I) ions*	*Pb(II) ions*	*Cd(II) ions*	*Ag(I) ions*	*Pb(II) ions*	*Cd(II) ions*
**K_2 (g mg^{-1} min $^{-1}$)**	0.00158	0.00061	0.00017	0.00762	0.000125	0.000174
**q_e (mg/g)**	147.059	136.986	102.041	138.889	72.993	69.93
**R_2**	0.9998	0.9999	0.9968	1	0.9891	0.9927

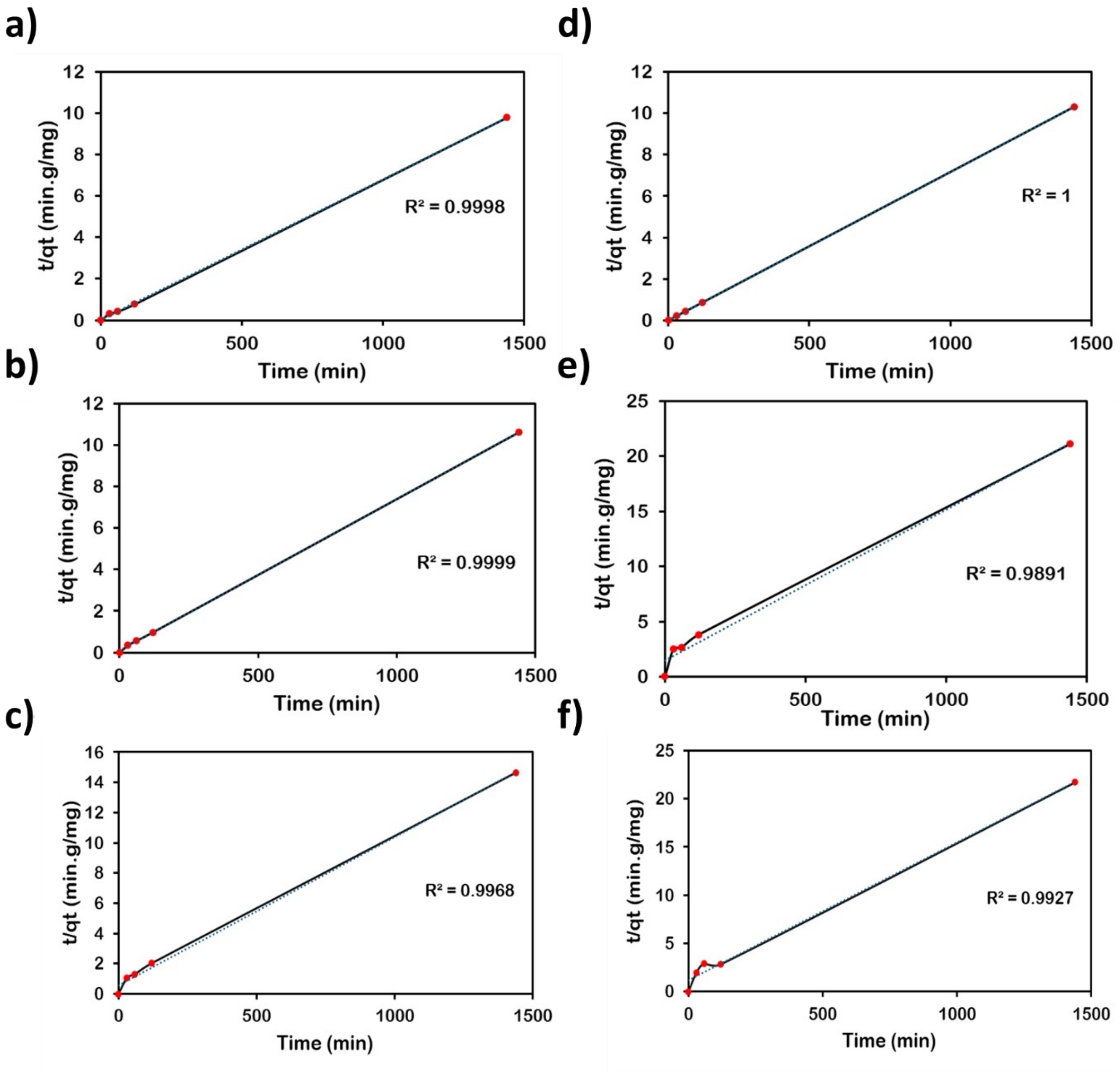

Figure S3: The pseudo-second-order kinetic plot for the adsorption of: a) ([Ag(I)] = 150 mg L−1) on a 10 mg AMCD, b) ([Pb(II)] = 150 mg L−1) on a 10 mg AMCD, c) ([Cd(II)] = 150 mg L−1) on a 10 mg AMCD, d) ([Ag(I)] = 150 mg L−1) on a 10 mg PVC-AMCD membrane, e) ([Pb(II)] = 150 mg L−1) on a 10 mg PVC-AMCD membrane, f) ([Cd(II)] = 150 mg L−1) on a 10 mg PVC-AMCD membrane.

4. Adsorption isotherm

Adsorption equilibrium experiments were carried out with different initial concentrations of **heavy metal** ions ranging between 100 and 800 mg/L to assess the interaction between the adsorbate (metal ions) and the adsorbent (AMCD and PVC-AMCD). 10 mg of both adsorbents were immersed in each of the prepared solutions and were left in the shaker at a speed of 100 rpm at room temperature for 30 minutes. Aliquots were taken from the solutions, filtered

separately through a 0.45 µm filter syringe and analyzed by AAS. The amount of adsorbed metal ion at equilibrium, qe (mg/g) was calculated by **EQN S2** [2]:

$$q_e = \frac{(C_i - C_e)v}{w} \qquad \textbf{(S2)}$$

Where C_i and C_e (mg/L) are the initial and equilibrium concentrations of metal ion, respectively. Volume of the solution V (L), and W is the mass of adsorbent used (g). The experimental data were fitted into Langmuir and Freundlich isotherm models [3–5], which are mathematical models that describe the distribution of the adsorbate and adsorbent in aqueous solution according to the heterogeneity/homogeneity of the adsorbent, coverage type, and interaction between the adsorbate species.

4.1. Langmuir isotherm model

The Langmuir equation verifies a monolayer interaction between the adsorbate molecules onto the surface of the adsorbent. The linear form of this isotherm is represented by the expression :[3]

$$\frac{C_e}{q_e} = \frac{C_e}{q_{max}} + \frac{1}{q_{max}k_L} \qquad \textbf{(S3)}$$

Where q_e (mg/g) and C_e (mg/L) are the amount of adsorbed metal ions per unit weight of adsorbent and concentration of metal ions in solution at equilibrium, respectively. The constant K_L (L/g) is the Langmuir equilibrium constant related to the energy of adsorption and q_{max} is maximum adsorption capacity (mg/g). The essential feature of the Langmuir isotherm can be expressed in terms of a dimensionless constant called separation factor (RL, also called equilibrium parameter) which is defined by the following equation:

$$R_L = \frac{1}{1 + K_L C_0} \qquad \textbf{(S4)}$$

Where C_0 (mg/L) is the initial adsorbate concentration. The value of R_L indicates the shape of the isotherms to be either unfavorable (R_L>1), linear (R_L=1), favorable (0<R_L<1) or irreversible (R_L=0).

4.2. Freundlich isotherm model

The most important multisite adsorption isotherm for heterogeneous surfaces is the Freundlich adsorption isotherm, and the linear form of this isotherm is expressed as:[5]

$$log\ q_e = log\ k_f + \frac{1}{n} log\ C_e \qquad \textbf{(S5)}$$

Where q_e is the metal uptake (mg/g) at equilibrium, K_F is the measure of the sorption capacity, 1/n is the sorption intensity, and C_e is the final ion concentration in solution, or equilibrium concentration (mg/L). The Freundlich isotherm constants K_F and 1/n are evaluated from the intercept and the slope, respectively of the linear plot of log q_e versus log C_e.

The value of n reflects the type of isotherm to be favorable ($0 < 1/n < 1$), irreversible ($1/n = 0$) or unfavorable ($1/n > 1$).

5. Regeneration and reusability of PVC-AMCD membrane

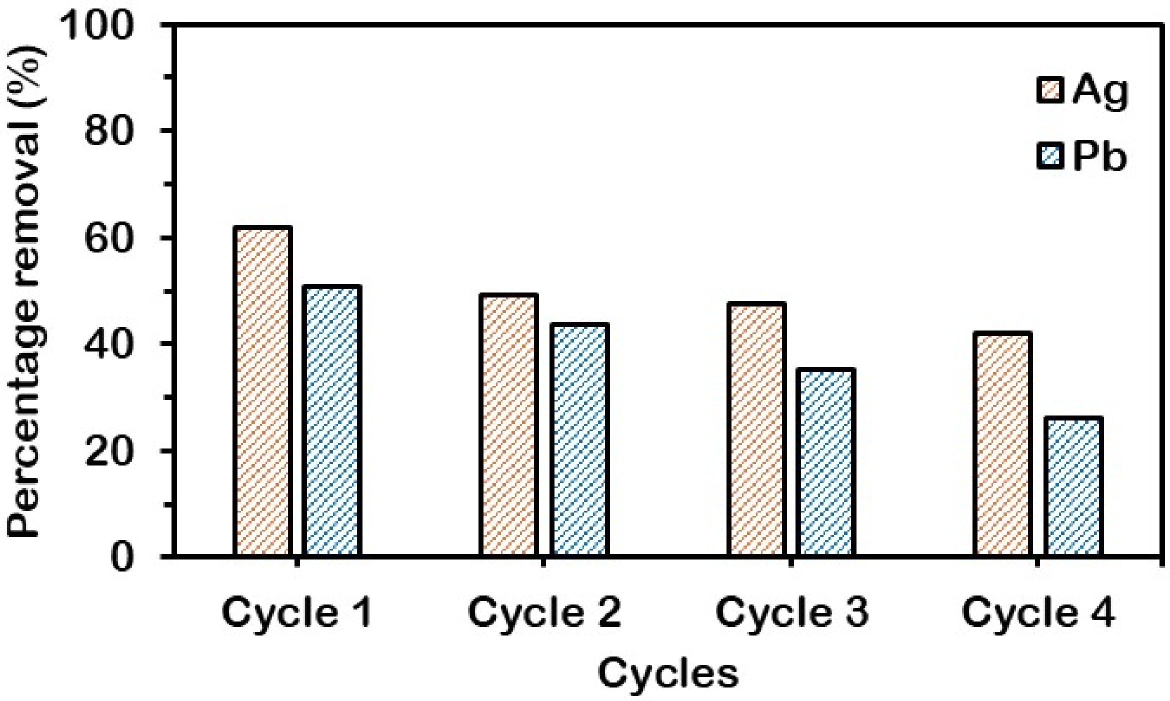

Figure S4: Reusability of PVC-AMCD for the adsorbed amount of Ag(I) and Pb(II).

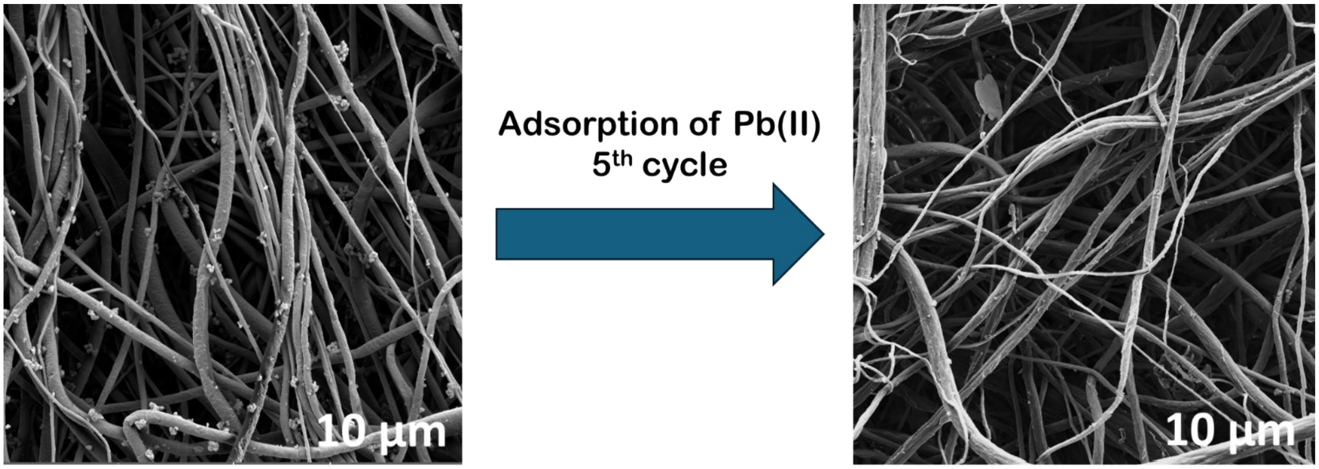

Figure S5: SEM images of PVC-AMCD membrane before and after regeneration.

6. Rapid filtration performance of mixed contaminants

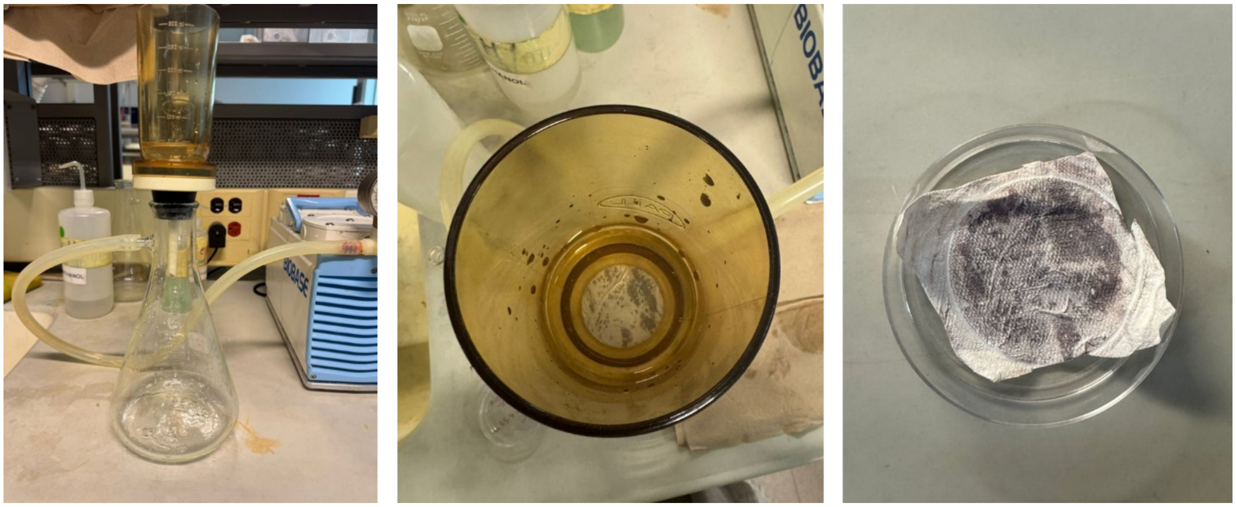

Figure S6: Filtration setup and PVC-AMCD membrane after filtration of multi-metal solution

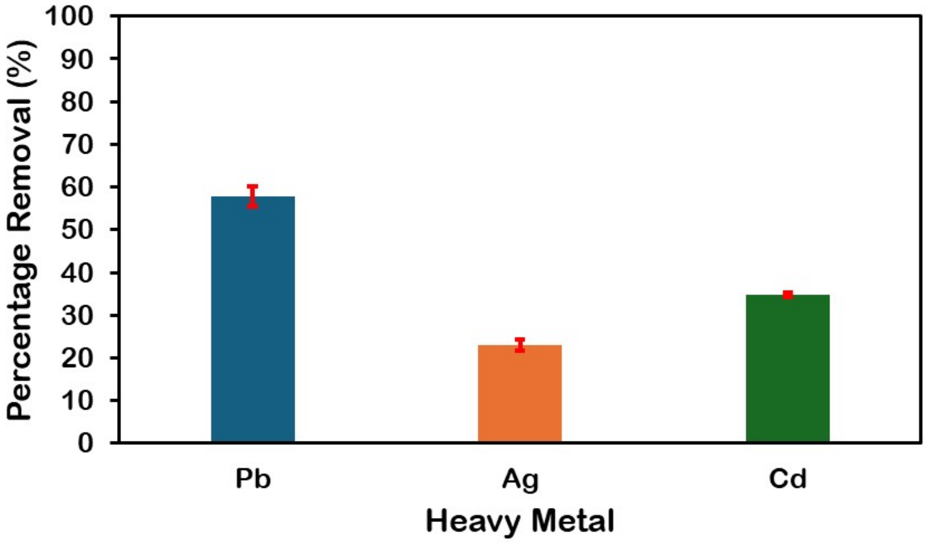

Figure S7: Percentage removal of 4x4 cm PVC-AMCD membrane during the filtration of a multi-metal solution. The solution was left in contact with the membrane for 10 minutes

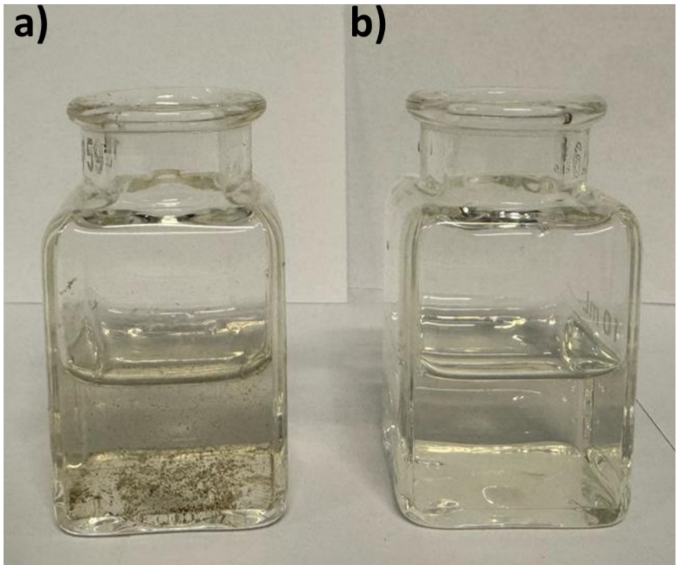

Figure S8: Real wastewater: a) before filtration and b) after filtration using PVC-AMCD membrane

7. Comparative analysis of adsorption capacities towards lead(II) ions

Table S3*:* Comparison of maximum lead(II) adsorption capacity qmax (mg g-1) of PVC-AMCD membrane with previously reported MOF/ZIF-based membranes

MOF / ZIF - based membrane	q_{max} (mg g-1)	pH	Time (min)	Reference
ZIF-8 membrane on alumina support	115.38	7	120	[6]

UiO-66-NH$_2$PAN/chitosan nanofibers	441.2	2–7	5–90	7
HKUST-1 MOF on polyethersulfone (PES)	–	–	60	8
UiO-66-(COOH)$_2$ in a PAN nanofibrous substrate (TFNC membrane, Ca-alginate skin)	254.5	–	–	9
UiO-66-(COOH)$_2$ / PVC electrospun membrane	203	–	–	10
ZIF-8/β-cyclodextrin in PVDF nanofibrous mixed-matrix membrane	708.13	–	–	11
ZIF-67/PVA Nanofibers Composite	140.3	6	120	12
MOF-on-nanofiber membranes (Fe/Zr-MOFs on PAN/PVDF) **MOF 800**	170.74	5	50	13
Versatile Mixed-Matrix Membranes Based on AMCD-ZIF and PVC for Sustainable Water Remediation	1666.7	7	30	This study

8. References

1 D. Yang, L. Li, B. Chen, S. Shi, J. Nie and G. Ma, *Polymer*, DOI:10.1016/j.polymer.2018.12.046.

2 K. T. Aung, this link will open in a new window Link to external site, H. Seung-Hee, S.-J. Park and L. Chang-Gu, *Applied Sciences*, 2020, **10**, 1738.

3 M. B. Desta, *Journal of Thermodynamics*, 2013, **2013**, e375830.

4 T. S. Khayyun and A. H. Mseer, *Appl Water Sci*, 2019, **9**, 170.

5 A. Mittal, L. Kurup and J. Mittal, *Journal of Hazardous Materials*, 2007, **146**, 243–248.

6 D. N. A. Chee, F. Aziz, A. F. Ismail, A. B. H. Kueh, M. A. M. Amin and M. Amran, *Chemical Engineering Science*, 2023, **276**, 118775.

7 S. Jamshidifard, S. Koushkbaghi, S. Hosseini, S. Rezaei, A. Karamipour, A. Jafari rad and M. Irani, *Journal of Hazardous Materials*, 2019, **368**, 10–20.

8 S. S. A. Al-Qaisi, A. Raisi and Q. F. Alsalhy, *Scientific Reports*, 2025, **15**, 14636.

9 T. Zhang, P. Li, S. Ding and X. Wang, *Journal of Hazardous Materials*, 2022, **424**, 127742.

10 M. H. Hashem, M. Hammoud, M. N. Ahmad and M. Hmadeh, *ACS Appl. Mater. Interfaces*, 2025, **17**, 16275–16286.

11 C. Chen, Q. Liu, W. Chen, F. Li, G. Xiao, C. Chen, R. Li and J. Zhou, *Separation and Purification Technology*, 2022, **292**, 120993.

12 S. Singh, P. N., B. Uppara, R. Varshney, N. Shehata, N. A. Khan, J. Joji, J. Singh and P. C. Ramamurthy, *ACS EST Water*, 2024, **4**, 4497–4509.
13 J. E. Efome, D. Rana, T. Matsuura and C. Q. Lan, *J. Mater. Chem. A*, 2018, **6**, 4550–4555.
